# Case of Pericarditis and Double Pleurisy, with Effusion

**Published:** 1864-05

**Authors:** E. S. Gaillard

**Affiliations:** Medical Inspector.


ART. V.—*Case of Pericarditis and Double Pleurisy, with Effusion. Reported by* Surg. E. S. GAILLARD, Medical Inspector.

I transmit the following clinical notes of a case of pericarditis and double pleurisy, with effusion, observed by me in General Hospital, No 1, Savannah, Georgia, at the period of my inspection in November last. I am indebted to Surgeon Wm. Gaston Bullock, in charge of this hospital, and to Assistant-Surgeon J. Haring, having immediate charge of the patient, for the notes presented:

Private J. C——, Company "K," 57th Regt. Ga. Vols., was admitted into General Hospital, No. 1, Savannah, Ga., on the 9th of November, 1863.

*The History* of the case was most unsatisfactory. No surgeon's report was sent with the patient, and he was not able to give a clear account of himself. All that could be ascertained was, that he had been sick about five weeks, previous to his admission into this hospital; was taken sick with fever, pains in his side, &c. About the third week of his sickness, he had commenced "to swell," and since that time had suffered much from shortness of breath; had coughed a good deal, but never expectorated much.

*State of Patient when Admitted.*—Patient is greatly exhausted; pulse not susceptible; skin cold; respiration thirty-six per minute; general anasarcous condition of the body. A more careful examination was deferred on account of his great exhaustion, which was partially attributed to his having travelled several miles in an ambulance. The patient's condition is nearly the same (two hours after his admission) as when first seen; no pulse to be felt at the wrist.

*Percussion* reveals flatness over his whole chest; most so on the right side anteriorly and over the præcordial region; the latter is unnaturally prominent.

*Auscultation* shows that the respiratory murmur is greatly diminished in both lungs. This condition amounts to an almost entire absence of the same, except in the upper-thirds; there is bronchial respiration over the whole of the posterior portion of the thorax. The heart's sounds are feeble and distant, scarcely perceptible; patient breathes with great difficulty; has an anxious, distressed expression of countenance; coughs but little, and does not expectorate; tongue is clean and moist.

*Progress of the Case.*—The morning following his admission, the patient appeared to be more comfortable; had slept well during a part of the night; pulse is perceptible, but extremely weak and intermittent, two or three beats being followed by a shorter or a longer pause; no proper estimate as to the frequency of the pulse per minute can be formed: the same irregularity exists in the heart's beat; respiration twenty-six per minute; no change in the respiratory sounds. For the next two days no *material* change took place in the patient's condition. He gained, however, some strength, his appetite improved, and his respiration became somewhat less difficult and distressing. From the 14th day of November, (the sixth since his admission,) his condition became rapidly worse. The skin became dry and hot; the pulse rose to one hundred and twenty, and remained very feeble; respiration thirty. Erysipelas made its appearance, first on the right side of the neck and face, and afterwards in his right foot and leg; the dyspnœa became very distressing; he had to be supported in a sitting posture; suffered with constant cough, without, however, any expectoration. He died early on the morning of the 17th of November, 1863.

*The Diagnosis* of the case was made out to be pericarditis, with double pleurisy. The symptoms exhibited by the feeble pulse, anxious expression, distant and feeble sounds of the heart, together with præcordial fullness and extended flatness on percussion, led at once to the suspicion of pericarditis, with effusion. A further, and more extended examination, discovered also double pleurisy, with effusion. *Post-mortem* examination verified this diagnosis.

*Treatment.*—Owing to the exhausted condition of the patient, a stimulating tonic and supporting treatment was adopted and mainly relied upon, though a few small doses of calomel, squills and digitalis were administered, together with vesication over the region of the heart.

*Post-Mortem Examination.*—Six hours after death, there was presented a general anasarcous condition of the body. Inspection of the chest showed apparent enlargement of the left side; measurement, however, proved the right side to be larger by nearly one inch, the deception of the eye being caused by the great prominence of the præcordial region.—Extensive and strong adhesions, both recent and old, existed in the pleuræ of both sides of the chest; also large quantities of lymph, resembling lumps of fat, with serous effusion in both pleuræ, the latter being much greater in the right than in the left side. Some engorgement existed in the posterior and lower portion of both lungs. The pericardium was largely distended with serum and immense quantities of coagulable lymph, some floating in the serum, other portions encasing the heart, like fat, which could be readily peeled off. No other signs of disease in the heart were discovered.

This case is interesting chiefly on account of its great rarity, and on account of the profuse deposit of the products of acute inflammation. However common it is to see all of the serous sacs of the body distended in anasarca, it is seldom, even in military hospitals, (where are congregated the subjects of unusual exposure,) to find two or more of the serous sacs of the body simultaneously invaded by a common disease; the degree of inflammatory action being identical in all and the specific results entirely similar. It may be proper to mention that at the period of my examination, (about one week before the death of the patient,) there was a marked absence of that usual harmony in the rational and physical indices, ordinarily observed under similar pathological conditions. There was no orthopnœa, and in the movements necessary to a careful examination of the patient, no dyspnœa was manifested.

